# Nephroureterectomy for upper tract urothelial carcinoma recurrence in bladder cancer patients treated with radical cystectomy: a multicentric propensity score matched analysis on predictors, practice patterns and survival outcomes

**DOI:** 10.1007/s00345-026-06520-z

**Published:** 2026-06-10

**Authors:** Valerio Santarelli, Vincenzo Asero, Roberta Corvino, Ettore De Berardinis, Alessandro Sciarra, Giuseppe Basile, Yasmin Abu-Ghanem, Rajesh Nair, Muhamad Shamin Khan, Ramesh Thurairaja, Wojciech Krajewski, Jan Laszkiewicz, Simone Albisinni, Keiichiro Mori, Felix Guerrero-Ramos, David D’Andrea, Andrea Gallioli, Ekaterina Laukhtina, Elisabeth Grobet-jeandin, Maxence Reynard, Andrea Mari, Laura S. Mertens, Francesco Soria, Jeremy Teoh, Francesco Claps, Antonio Amodeo, Aleksander Ślusarczyk, Roberto Contieri, Sisto Perdonà, José Daniel Subiela, Karl H. Tully, Renate Pichler, Alessandro Antonelli, Riccardo Bertolo, Francesco Ditonno, Rodolfo Hurle, Felice Crocetto, Roman Mayr, Angelo Porreca, Filippo Marino, Giuseppe Simone, Gabriele Tuderti, Bernardo Maria Cesare Rocco, Chiara Sighinolfi, Luigi Vittori, Marco Moschini, Paolo Zaurito, Mario De Angelis, Pietro Scillipoti, Luca Afferi, Ruben De Groote, Geert De Naeyer, Alexandre Mottrie, Nicola Pavan, Benjamin I. Chung, Gautier Marcq, Benjamin Pradere, Francesco Del Giudice

**Affiliations:** 1 Department of Maternal Infant and Urologic Sciences, Policlinico Umberto I Hospital, “Sapienza” University of Rome, Rome, Italy; 2 Orsi Academy, Melle, Belgium; 3 Department of Urology, Stanford University School of Medicine, Stanford, USA; 4 Department of Urology, Royal Free London Foundation Trust, London, UK; 5 Guy’s and St. Thomas’ NHS Foundation Trust, Guys and St. Thomas’ Hospital, London, UK; 6 Department of Minimally Invasive and Robotic Urology, University Center of Excellence in Urology, Wroclaw Medical University, 50-556 Wroclaw, Poland; 7 Unit of Urology, Department of Surgical Sciences, Tor Vergata University, Rome, Italy; 8 Department of Urology, The Jikei University School of Medicine, Tokyo, Japan; 9 Department of Urology, Hospital Universitario 12 de Octubre, Madrid, Spain; 10 Department of Urology, Comprehensive Cancer Center, Medical University of Vienna, Vienna, Austria; 11 Department of Urology, Fundació Puigvert, Barcelona, Spain; 12 Department of Surgery, Autonomous University of Barcelona, Barcelona, Spain; 13 Division of Urology, Geneva University Hospitals, Geneva, Switzerland; 14 Unit of Oncologic Minimally-Invasive Urology and Andrology, Department of Clinical and Experimental Medicine, Careggi Hospital, University of Florence, Florence, Italy; 15 Department of Urology, The Netherlands Cancer Institute, Amsterdam, The Netherlands; 16 Surgical Sciences, University of Turin and Città della Salute e della Scienza, Turin, Italy; 17 Department of Surgery, S.H. Ho Urology Centre, The Chinese University of Hong Kong, Hong Kong, Hong Kong; 18 Veneto Institute of Oncology IOV - IRCCS, Padua, Italy; 19 Department of General, Oncological and Functional Urology, Medical University of Warsaw, Warsaw, Poland; 20 Department of Urology, National Cancer Institute, IRCCS Fondazione G. Pascale, Naples, Italy; 21 Department of Urology, Hospital Universitario Ramón y Cajal, Instituto Ramón y Cajal de Investigación Sanitaria, Universidad de Alcala, Madrid, Spain; 22 Department of Urology and Neurourology, Marien Hospital Herne, Ruhr-University Bochum, Bochum, Germany; 23 Department of Urology, Comprehensive Cancer Centre Innsbruck, Medical University of Innsbruck, Innsbruck, Austria; 24 Department of Urology, University of Verona, Azienda Ospedaliera Universitaria Integrata, Verona, Italy; 25 Department of Urology, Humanitas Clinical and Research Hospital, IRCCS, Milano, Italy; 26 Department of Neurosciences, Reproductive Sciences and Odontostomatology, University of Naples Federico II, Naples, Italy; 27 Department of Urology, University Hospital Augsburg, Augsburg, Germany; 28 Department of Urology, Humanitas Gavazzeni, 24125 Bergamo, Italy; 29 Department of Urology, IRCCS Regina Elena National Cancer Institute, Rome, Italy; 30 Department of Urology, ‘A. Gemelli’ Hospital Foundation, IRCCS, Università Cattolica del Sacro Cuore, Rome, Italy; 31 Department of Radiological, Oncological and Pathological Sciences, Sapienza University of Rome, Rome, Italy; 32 Department of Urology, IRCCS San Raffaele Hospital, Milan, Italy; 33 Department of Urology, Luzerner Kantonsspital, Lucerne, Switzerland; 34 Azorg Hospital, Aalst, Belgium; 35 Department Me.Pre.Cc, Precision Medicine in Medical, Surgical, and Critical Areas, Section of Urology, University of Palermo, Palermo, Italy; 36 Department of Urology, Claude Huriez Hospital, CHU Lille, Lille, France; 37 Department of Urology, St. Josef Medical Center, University of Regensburg, Regensburg, Germany

**Keywords:** Radical cystectomy, Radical Nephroureterectomy, Upper tract urothelial carcinoma, Urothelial recurrence, Bladder cancer

## Abstract

**Aim:**

(I) To determine clinicopathological determinants of metachronous Upper Tract Urothelial Carcinoma (UTUC) requiring Radical Nephroureterectomy (RNU) after Radical Cystectomy (RC). (II) To evaluate long-term survival of patients who underwent RC + RNU compared with matched RC only controls.

**Methods:**

Patients undergoing RNU for metachronous UTUC were extracted from a multi-institutional RC database. A 1:2 Propensity Score Match (PSM) was performed based on age, gender, BMI, CCI, Smoking Status, and cT stage between RC only and RC + RNU patients. Simon-Makuch plots, landmark analyses and Multivariable Cox regressions were adopted to compare survival outcomes.

**Results:**

Of 1804 RC patients, 85 (4.7%) underwent subsequent RNU. At multivariate regression, younger age, smoking history, BCG exposure, NMIBC, CIS and positive ureteric margins were identified as positive predictors of upper tract recurrence. After PSM, 81 RC + RNU patients were matched to 157 RC only controls. Median time from RC to RNU was 29 (18–47) months, with the majority of UTUC diagnosed at a muscle-invasive stage (70.9%). Simon-Makuch curves demonstrated worse Cancer Specific Survival (CSS) of the RC + RNU cohort (HR: 6.41, 95% CI 3.16–13.04). At landmark analyses, RNU was consistently associated with an increased mortality risk from the 24th month onward. Multivariable Cox regression identified RNU and pN + as the only significant predictors of worse CSS (respectively, HR: 6.55, 95% CI 2.93–14.64 and HR: 8.45, 95% CI 3.24–21.99).

**Conclusions:**

The worse survival outcomes and high rates of locally advanced disease found at RNU underscore the need for standardized, risk-stratified, long-term follow-up of the remnant urinary tract following RC.

**Supplementary Information:**

The online version contains supplementary material available at 10.1007/s00345-026-06520-z.

## Introduction

Upper Urinary Tract (UUT) recurrence in the remnant urothelium represents a persistent, life-long risk following Radical Cystectomy (RC) [1]. Accordingly, latest European Association of Urology (EAU) guidelines suggest stringent UUT follow-up schedules for BC patients, with 4–10% of RC patients developing an Upper Urinary Tract Urothelial Carcinoma (UTUC). Radical nephroureterectomy (RNU) is the standard of care for high-risk UTUC [2, 3]. However, RNU following RC represents an additional burden in the already demanding treatment pathway for urothelial disease.

Notably, only few risk factors for UUT recurrence have been previously identified [1, 4]. Among these, bladder carcinoma in situ (CIS), multifocal disease, non-muscle invasive bladder cancer (NMIBC), and involvement of the distal ureter or prostatic urethra are recognized as the most prominent predictors for secondary UTUC [4]. Beyond these pathology-based features, limited evidence exists regarding the influence of surgical approach during RC, type of urinary diversion, and patient-related risk factors.

Despite the well-accepted recurrence risk, an effective standardized and risk-stratified follow-up regimen after RC for BC is still lacking [5]. Particularly, no consensus was reached regarding the timing of urinary cytology or the role of cross-sectional imaging in detecting late recurrences (i.e., beyond 5 years), which frequently involve the UUT [6, 7]. Besides its direct impact on cancer-specific survival outcomes, UTUC recurrence and its subsequent treatment further compromise overall health status primarily by worsening renal function and ultimately increasing cardiovascular events and all-cause deaths [2, 5, 6].

To date, real-world evidence on non-pathological determinants of UTUC recurrence after RC remains scarce. Moreover, limited data are available regarding the impact of RNU on survival outcomes of RC patients. To address this gap, we specifically selected patients who underwent RC for BC and subsequently RNU for an UUT recurrence to evaluate clinical and pathological determinants of UUT recurrence. After Propensity Score Matching on selected preoperative clinical characteristics, long-term survival outcomes were finally evaluated.

## Materials and methods

### Study population

Data from 23 tertiary referral centers in Europe, North America and Asia on patients who underwent RC with curative intent from 2005 to 2024 was collected from a multi-institutional, prospectively maintained database of the Young Academic Urologists (YAU)—Urothelial Carcinoma Working Group. Subsequently, the same dataset was retrospectively reviewed to extract patients who developed a UUT recurrence requiring RNU. Before study initiation, data-transfer agreement and Institutional Review Boards (IRB) approval from each of the included centers were obtained. All patient-related information was anonymized prior to data sharing. Exclusion criteria were previous Radiation Therapy or Chemotherapy performed for other purposes other than a Neoadjuvant/Adjuvant setting, RC performed for other indications rather than curative intent for a cN0M0 BC, estimated life expectancy < 5 years and concomitant or previous RNU.

### Statistical analysis

Statistical analyses were conducted using the R software environment for statistical computing and graphics (R version 4.2.2, R Foundation for Statistical Computing, Vienna, Austria). Baseline pertinent study cohort characteristics were summarized with descriptive statistics. Multivariable binary regression model was constructed to determine clinical and pathological determinants of UUT recurrence following RC. To balance baseline characteristics between the RC alone and the RC + RNU cohorts, a 1:2 Propensity Score Match (PSM) analysis using the nearest neighbour method with a calliper size of 0.2 was performed based on age, gender, BMI, CCI, Smoking Status, and Clinical T Stage. Relevant study information and outcomes of the matched cohorts were subsequently reported stratified according to RNU status (no RNU vs. RNU). To account for the time-dependent impact of performing RNU on long-term survival status, univariate survival analysis was performed using the Simon-Makuch variant of Kaplan–Meier plots and landmark analyses. Survival for all time-to-event analyses was defined as the time between radical cystectomy and death or last patient contact. Cox proportional hazards regression was used to estimate Hazard Ratio (HR) with 95% Confidence Interval (CI). Finally, multivariable Cox regression models with RNU-status as a time-dependent covariate were constructed for both Cancer Specific Survival (CSS) (defined as any death from urothelial cancer) and Overall Survival (OS).

## Results

### Descriptive statistics of the study population

In total, 1804 patients were identified (supplementary Table 1). Of these, 85 developed UTUC requiring RNU. Patients who underwent RNU after RC for a metachronous UTUC were more likely to undergo RC for a NMIBC diagnosis (67.1% vs. 31.5%, p < 0.001) and with a concomitant Carcinoma in Situ (CIS) (43.5% vs. 17.9%, p < 0.001). The majority of RC + RNU patients were previously exposed to intravesical BCG (n = 44, 51.8%), compared to only n = 317, 18.4% in the RC only cohort (p < 0.001). A pN + status at RC was more frequent in the RC alone group (n = 331, 18.1% vs. n = 2, 2.4%).

After 1:2 PSM, 81 RC + RNU patients were successfully matched with 157 patients in the RC alone group, meaning that n = 4 RC + RNU patients did not find a suitable match, and n = 5 were matched with a single RC alone patient. Characteristics of the matched cohort are reported in Table 1. Despite the similar NMIBC rates after matching on preoperative cT stage, patients who developed an UUT recurrence treated by RNU remained more frequently exposed to BCG (53.8% vs. 28.7%, p < 0.001). At final pathological analysis, RC alone patients were more likely to achieve a pT0 stage (no evidence of residual tumour, 26.1% vs. 6.2%), despite the similar NAC rates (23.6% vs. 17.3%, p = 0.321). However, MIBC rates at final RC specimens were similar between the two groups (46.5% vs. 43.2%, p = 0.713). As expected, patients who developed a subsequent RNU were more likely to present a positive ureteric margin at the final RC histopathological report (0.6% vs. 8.6%, p = 0.004). After a significantly longer follow-up (61.0 months [IQR 31.0–89.0] vs. 26.0 [6.0–60.0], p < 0.001), only 50%, n = 40 of RC + RNU were still alive, vs. 79.6%, n = 125 RC only patients, p < 0.001.

**Table 1:** General characteristics of the study population after propensity score matching

Variable	Overall N = 238	RC Only (N = 157)	RC + RNU (N = 81)	p-value
Age, median (IQR)	68.0 (60.0–73.0)	67.0 (58.0–74.0)	68.8 (60.8–72.9)	0.642
Gender, n (%)				0.543
Male	180 (75.6%)	116 (73.9%)	64 (79.0%)	
Female	58 (24.4%)	41 (26.1%)	17 (21.0%)	
BMI, median (IQR)	26.3 (23.5–29.0)	26.3 (23.3–28.4)	26.4 (24.0–30.0)	0.246
Charlson Comorbidity Index, median (IQR)	5.0 (3.0–6.0)	5.0 (3.0–6.0)	5.0 (3.0–6.0)	0.398
Smoking Status, n (%)				0.901
No	59 (24.8%)	40 (25.5%)	19 (23.5%)	
Active/Former	179 (75.2%)	117 (74.5%)	62 (76.5%)	
BCG exposure, n (%)				< 0.001
No	150 (62.9%)	112 (71.3%)	38 (46.3%)	
Yes	88 (37.1%)	45 (28.7%)	43 (53.8%)	
MIBC at TURBT, n (%)				0.212
No	138 (58.1%)	86 (54.8%)	52 (64.6%)	
Yes	100 (41.9%)	71 (45.2%)	29 (35.4%)	
Clinical T stage (cT), n (%)				0.893
cT0	0 (0.0%)	0 (0.0%)	0 (0.0%)	
cT1	98 (41.2%)	64 (40.8%)	34 (42.0%)	
cT2	95 (39.9%)	64 (40.8%)	31 (38.3%)	
cT3	6 (2.5%)	4 (2.5%)	2 (2.5%)	
cT4	7 (2.9%)	3 (1.9%)	4 (4.9%)	
cTis	16 (6.7%)	11 (7.0%)	5 (6.2%)	
cTa	16 (6.7%)	11 (7.0%)	5 (6.2%)	
CIS at TURBT, n (%)				< 0.001
No	173 (72.8%)	128 (81.5%)	45 (55.1%)	
Yes	65 (27.2%)	29 (18.5%)	36 (44.9%)	
Clinical N stage (cN), n (%)				0.936
cN0	234 (98.3%)	154 (98.1%)	80 (98.7%)	
cN +	4 (1.7%)	3 (1.9%)	1 (1.3%)	
Neoadjuvant Chemotherapy, n (%)				0.321
No	187 (78.6%)	120 (76.4%)	67 (82.7%)	
Yes	51 (21.4%)	37 (23.6%)	14 (17.3%)	
Urinary diversion, n (%)				0.064
Ileal conduit	131 (55.0%)	81 (51.6%)	50 (61.7%)	
Neobladder	70 (29.4%)	49 (31.2%)	21 (25.9%)	
Cutaneous ureterostomy	35 (14.7%)	27 (17.2%)	8 (9.9%)	
Ileal Pouch	2 (0.8%)	0 (0.0%)	2 (2.5%)	
Pathologic T stage, n (%)				< 0.001
pT0	46 (19.3%)	41 (26.1%)	5 (6.2%)	
pT1	40 (16.8%)	18 (11.5%)	22 (27.2%)	
pT2	42 (17.6%)	21 (13.4%)	21 (25.9%)	
pT3	49 (20.6%)	41 (26.1%)	8 (9.9%)	
pT4	17 (7.1%)	11 (7.0%)	6 (7.4%)	
pTis	34 (14.3%)	19 (12.1%)	15 (18.5%)	
pTa	10 (4.2%)	6 (3.8%)	4 (4.9%)	
MIBC at RC, n (%)				0.713
NMIBC	130 (54.6%)	84 (53.5%)	46 (56.8%)	
MIBC	108 (45.4%)	73 (46.5%)	35 (43.2%)	
pN stage at RC, n (%)				0.010
pN0	214 (89.9%)	135 (86.0%)	79 (97.5%)	
pN +	24 (10.1%)	22 (14.0%)	2 (2.5%)	
CIS at RC				0.385
No	147 (61.8%)	101 (64.3%)	46 (56.8%)	
Yes	91 (38.2%)	56 (35.7%)	35 (43.2%)	
Variant Histology at RC, n (%)				0.767
No	204 (85.6%)	134 (85.4%)	70 (86.1%)	
Yes	34 (14.4%)	23 (14.6%)	11 (13.9%)	
Ureteral Margin, n (%)				0.004
Negative	230 (96.6%)	156 (99.4%)	74 (91.4%)	
Positive	8 (3.4%)	1 (0.6%)	7 (8.6%)	
Time to RNU, median (IQR)	29.0 (18.0–47.0)	NA (NA-NA)	29.0 (18.0–47.0)	
Neoadjuvant CHT before RNU, n (%)				
No	76 (93.8%)	0 (NA%)	76 (93.8%)	
Yes	5 (6.2%)	0 (NA%)	5 (6.2%)	
Clinical T stage at RNU, n (%)				
cT0	0 (0.0%)	0 (NA%)	0 (0.0%)	
cT1	16 (20.8%)	0 (NA%)	16 (20.8%)	
cT2	32 (41.6%)	0 (NA%)	32 (41.6%)	
cT3	16 (20.8%)	0 (NA%)	16 (20.8%)	
cT4	4 (5.2%)	0 (NA%)	4 (5.2%)	
cTis	2 (2.6%)	0 (NA%)	2 (2.6%)	
cTa	3 (3.9%)	0 (NA%)	3 (3.9%)	
cTx	4 (5.2%)	0 (NA%)	4 (5.2%)	
Clinical N stage RNU, n (%)				
cN0	69 (86.3%)	0 (NA%)	69 (86.3%)	
cN +	11 (13.8%)	0 (NA%)	11 (13.8%)	
Tumor location RNU, n (%)				
Ureter	28 (34.6%)	0 (NA%)	28 (34.6%)	
Pelvicalyceal system	37 (45.7%)	0 (NA%)	37 (45.7%)	
Both	16 (19.8%)	0 (NA%)	16 (19.8%)	
Pathologic T stage RNU, n (%)				
pT0	5 (6.3%)	0 (NA%)	5 (6.3%)	
pT1	18 (22.8%)	0 (NA%)	18 (22.8%)	
pT2	21 (26.6%)	0 (NA%)	21 (26.6%)	
pT3	28 (35.4%)	0 (NA%)	28 (35.4%)	
pT4	7 (8.9%)	0 (NA%)	7 (8.9%)	
Pathologic N stage RNU, n (%)				
pN0	32 (40.0%)	0 (NA%)	32 (40.0%)	
pN1	4 (5.0%)	0 (NA%)	4 (5.0%)	
pN2	10 (12.5%)	0 (NA%)	10 (12.5%)	
pN3	2 (2.5%)	0 (NA%)	2 (2.5%)	
pNx	32 (40.0%)	0 (NA%)	32 (40.0%)	
VH RNU, n (%)				
No	72 (88.9%)	0 (NA%)	72 (88.9%)	
Yes	9 (11.1%)	0 (NA%)	9 (11.1%)	
LVI RNU, n (%)				
No	55 (67.9%)	0 (NA%)	55 (67.9%)	
Yes	26 (32.1%)	0 (NA%)	26 (32.1%)	
CIS RNU, n (%)				
No	54 (66.7%)	0 (NA%)	54 (66.7%)	
Yes	27 (33.3%)	0 (NA%)	27 (33.3%)	
Adjuvant CHT RNU, n (%)				
No	69 (85.2%)	0 (NA%)	69 (85.2%)	
Yes	12 (14.8%)	0 (NA%)	12 (14.8%)	
Last FU, months, median (IQR)	34.5 (12.0–63.0)	26.0 (6.0–60.0)	61.0 (31.0–89.0)	< 0.001
Follow-Up Status, n (%)				< 0.001
Alive	165 (69.6%)	125 (79.6%)	40 (50.0%)	
Dead of disease	48 (20.3%)	23 (14.6%)	25 (31.3%)	
Dead of other causes	24 (10.1%)	9 (5.7%)	15 (18.8%)	

*RC* radical cystectomy, *RNU* radical nephroureterectomy, *IQR* interquartile range, *BMI* body mass index, *BCG* bacillus calmette-guérin, *MIBC* muscle-invasive bladder cancer, *TURBT* transurethral resection of bladder tumor, *CIS* carcinoma in situ, *NMIBC* non-muscle-invasive bladder cancer, *CHT* chemotherapy, *VH* variant histology, *LVI* lymphovascular invasion, *FU* follow-up, *NA* not applicable

### Risk factors for UUT recurrence requiring RNU in the unmatched cohort

Results of multivariable binary logistic regression analysis exploring predictors of UUT recurrence requiring RNU among patients submitted to RC for BC are reported in Table 2. Older age reduced the risk of a subsequent RNU (Adjusted Odds Ratio (aOR): 0.940, 95% CI 0.921–0.959, p < 0.001). History of smoking (either former or active) and BCG exposure were significantly associated with an increasing risk of RNU (aOR: 1.81, 95% CI 1.012–3.277, p = 0.046 for smoking status and aOR: 2.12, 95% CI 1.21–5.77, p = 0.021 for BCG exposure). Regarding histological determinants, NMIBC, concomitant CIS, and positive ureteric margin were all significant independent predictors of UUT recurrence (respectively aOR: 4.67, 95% CI 2.68–8.13, p < 0.001; aOR: 3.49, 95% CI 2.11–5.79, p < 0.001 and aOR 10.71, 95% CI 2.97–38.60, p < 0.001). Finally, the Urinary Diversion of choice, gender, preoperative NAC and RC approach had no significant impact on the risk of UTUC development in RC patients.

**Table 2:** Multivariable binary regression exploring risk factors for UTUC development in the unmatched cohort

Variable	aOR	95% CI	p value
Age	0.94	0.92–0.96	< 0.001
Former/Active Smoker	1.82	1.01–3.23	0.046
Gender (female)	1.01	0.59–2.03	0.782
NAC	0.87	0.45–1.69	0.679
BCG exposure	2.12	1.21–5.77	0.021
RC approach	1.02	0.41–2.14	0.863
NMIBC	4.67	2.68–8.13	< 0.001
Variant Histology	1.28	0.61–2.67	0.506
CIS	3.49	2.11–5.79	< 0.001
Ureteral Margin	10.71	2.97–38.60	< 0.001
Urinary Diversion (UCS vs. IC)	0.93	0.44–1.96	0.859
Urinary Diversion (NB vs. IC)	0.43	0.161–1.12	0.084

*aOR* adjusted odds ratio, *CI* confidence interval, *NAC* neoadjuvant chemotherapy, *BCG* bacillus calmette-guérin, *RC* radical cystectomy, *NMIBC* non-muscle-invasive bladder cancer, *CIS* carcinoma in situ, *UCS* cutaneous ureterostomy, *IC* ileal conduit, *NB* neobladder

### Survival outcomes by treatment group

#### Overall survival

**Fig. 1:**
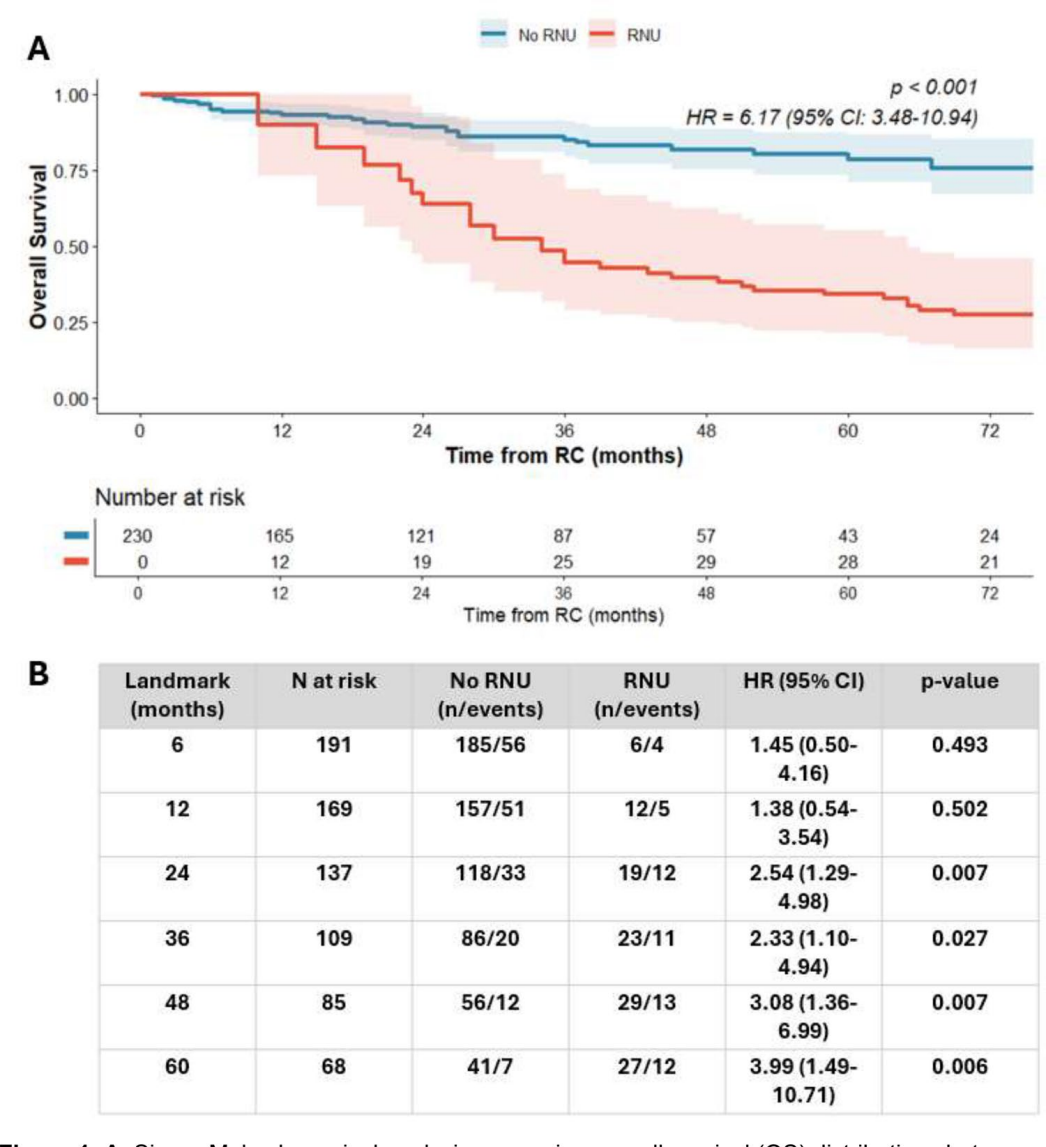
**A** Simon-Makuch survival analysis comparing overall survival (OS) distributions between the RC alone group and the RC + RNU group in the matched cohort; **B** Landmark analysis for OS according to RNU status (matched cohort)

Simon-Makuch survival analyses were carried out to compare OS distributions between patients who underwent RC alone vs. those who subsequently required RNU both in the matched and unmatched cohorts (supplementary Fig. 1a and Fig. 1a). RNU was treated as a time-dependent covariate, allowing patients to contribute to the no-RNU group until the time of RNU and subsequently to the RNU group. In the matched cohort, Restricted Mean Survival Time (RMST) was 133.9 months (SE = 5.79) for the RC alone group and 53.3 months (SE = 8.44) for the RC + RNU group. Results of Cox proportional hazards regression with RNU as a time-dependent covariate showed a significantly higher risk of all-cause death in patients requiring RNU (HR = 6.17, 95% CI 3.48–10.94, p < 0.001).

Landmark analyses were performed at 6, 12, 24, 36, 48 and 60 months both in the unmatched (supplementary Fig. 1b) and matched (Fig. 1b) cohorts, to assess the association between RNU and OS. Classification of the patients varied at each landmark based on RNU status (no RNU vs. RNU). In both cohorts, RNU was not significantly associated with worse OS at early landmarks (6 and 12 months). However, from 24 months onwards, RNU was consistently associated with a significantly increased overall mortality risk, reaching an HR of 3.99 (95% CI 1.49–10.71, p = 0.006) at 60 months.

#### Cancer specific survival

**Fig. 2:**
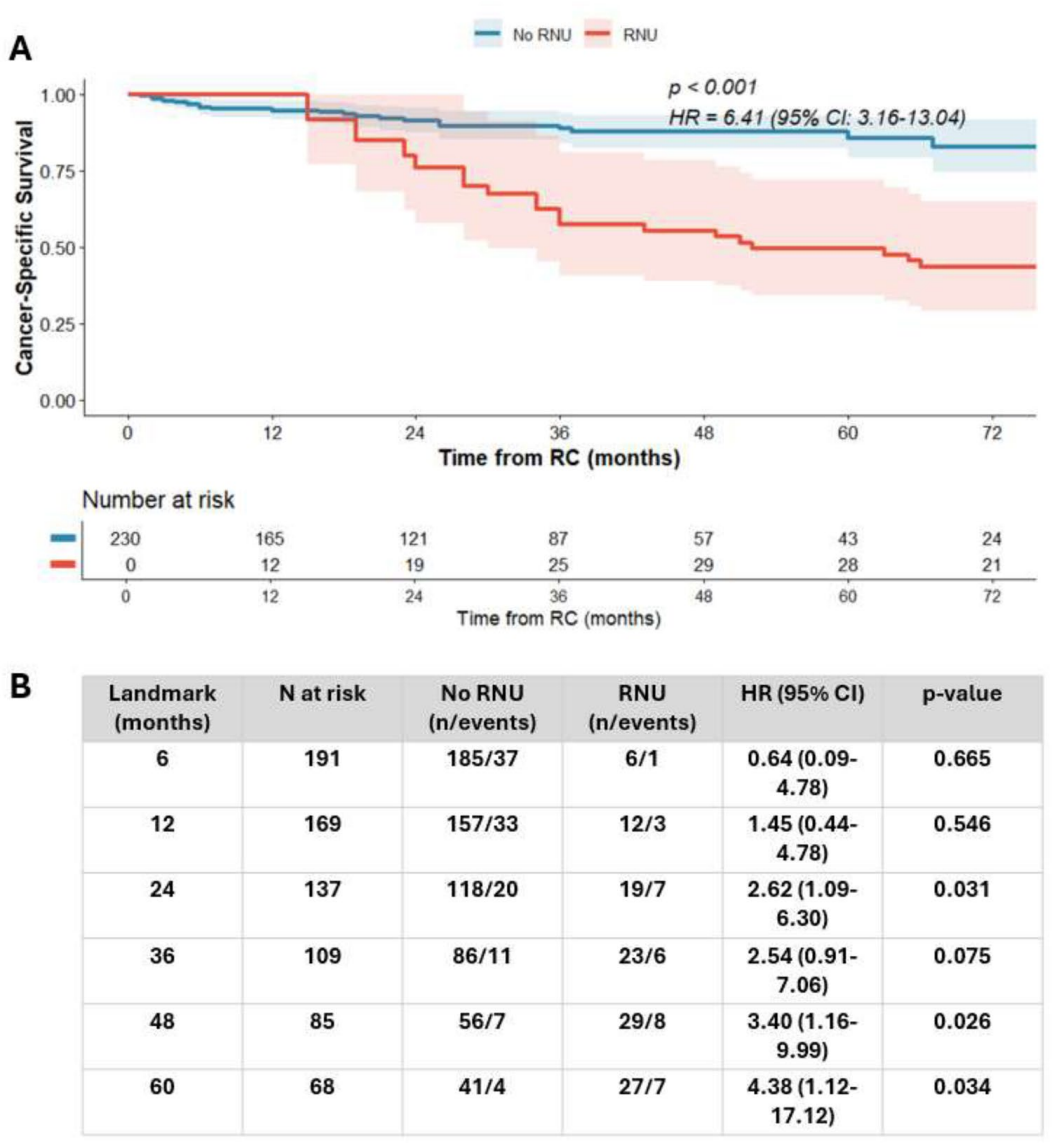
**A** Simon-Makuch survival analysis comparing Cancer Specific Survival (CSS) distributions between the RC alone group and the RC + RNU group in the matched cohort; **B** Landmark analysis for CSS according to RNU status (matched cohort)

Supplementary Fig. 2a and Fig. 2a depict results of Simon-Makuch survival analysis comparing CSS distributions in the unmatched and matched cohorts, respectively. After 1:2 PSM, RMST was 143.4 months (SE = 5.23) for the RC alone group and 75.9 months (SE = 11.03) for the RC + RNU group after PSM. Results of Cox proportional hazards regression with RNU as a time-dependent covariate showed a significantly higher risk of cancer-related death in patients requiring RNU (HR = 6.41, 95% CI 3.16–13.04, p < 0.001).

The temporal effect of RNU on CSS was assessed with Landmark analyses carried out at 6, 12, 24, 36, 48 and 60 months before and after PSM (Supplementary Fig. 2b and Fig. 2b). Similarly to OS, RNU was not significantly associated with worse CSS at early landmarks (6 and 12 months). Subsequently, the impact of RNU on CSS increased, reaching statistical significance at 24 months (HR = 2.62, 95% CI 1.09–6.30, p = 0.031), 36 months (HR: 2.54, 95% CI 0.91–7.06, p = 0.075), 48 months (HR = 3.40, 95% CI 1.16–9.99, p = 0.026), and 60 months (HR = 4.38, 95% CI 1.12–17.12, p = 0.034).

### Multivariable predictors of survival outcomes

Cox proportional hazard regression analyses with RNU as a time-dependent covariate were performed to identify predictors of OS and CSS in the matched cohort (Table 3). Undergoing RNU after RC was independently associated with a significantly increased risk of both all-cause and cancer-related death (HR 5.44, 95% CI 2.89–10.24, p < 0.001 for OS and HR: 6.55, 95% CI 2.93–14.64, p < 0.001 for CSS). LN involvement at RC was also a strong independent predictor of mortality in both models (HR: 6.25, 95% CI 2.78–14.04, p < 0.001 for OS and HR: 8.45, 95% CI 3.24–21.99, p < 0.001 for CSS). Older age was significantly associated with worst OS (HR 1.05 per year, 95% CI 1.02–1.08, p < 0.001) but not with CSS (HR 1.03, 95% CI 0.99–1.06, p = 0.143). Gender, smoking status, MIBC at TURBT, CIS in the bladder, histology at TURBT, pathological T stage at RC, and type of urinary diversion were not significantly associated with either OS or CSS (p > 0.05).

**Table 3:** Multivariable cox regression analysis with RNU as a time-dependent covariate exploring independent predictors of overall survival (OS) and cancer specific survival (CSS)

	OS			CSS		
Variable	HR	95% CI	p-value	HR	95% CI	p-value
RNU	5.44	2.89–10.24	< 0.001	6.55	2.93–14.64	< 0.001
Age	1.05	1.02–1.08	< 0.001	1.03	0.99–1.06	0.143
Gender (female)	0.73	0.38–1.41	0.350	0.44	0.18–1.07	0.069
Smoking Status	0.87	0.46–1.64	0.657	0.68	0.32–1.43	0.306
MIBC at TURBT	1.00	0.57–1.75	0.999	1.14	0.58–2.23	0.709
CIS in the bladder	0.94	0.54–1.64	0.834	0.87	0.43–1.78	0.709
Histology at RC	1.36	0.70–2.65	0.363	1.09	0.47–2.53	0.839
pT RC	1.26	0.71–2.23	0.425	1.43	0.70–2.89	0.325
pN RC (positive)	6.25	2.78–14.04	< 0.001	8.45	3.24–21.99	< 0.001
Urinary diversion	1.20	0.87–1.64	0.262	0.97	0.65–1.45	0.894

*OS* overall survival, *CSS* cancer-specific survival, *HR* hazard ratio, *CI* confidence interval, *RNU* radical nephroureterectomy, *MIBC* muscle-invasive bladder cancer, *TURBT* transurethral resection of bladder tumor, *CIS* carcinoma in situ, *RC* radical cystectomy

Finally, a subgroup analysis was performed to identify UTUC-related predictors of OS and CSS among patients who underwent RNU after RC (Supplementary Table 2). Positive lymph nodes and Variant histology were the only independent predictors of survival outcomes (respectively, HR: 2.19, 95% CI 1.00–6.72, p = 0.049 and HR: 4.12, 95% CI 1.56–10.89, p = 0.004 for OS; HR: 3.96, 95% CI 1.54–10.14, p = 0.004 and HR: 4.30, 95% CI 1.29–14.33, p = 0.017 for CSS).

## Discussion

The majority of systemic BC recurrences typically develop within the first two years following RC, while urothelial recurrence within the remnant UUT has been reported to develop throughout a patient’s lifetime [8–12]. In our multicenter cohort of 1804 RC patients, 4.71% developed an UUT recurrence requiring RNU. Considering that RNU is the standard of care for high-risk UTUC, and a rate of 61% of UUT recurrences after RC managed with RNU reported by a contemporary review of post-RC UTUC management, our results are in line with the available literature[13–15]. In light of the prognostic advantage of early diagnosis for UTUC, current EAU guidelines suggest CT scan every six months until the third year, followed by annual imaging thereafter [13]. In the present study, we reported a median time from RC to RNU of 29 months, with an IQR of 18 to 47 months. These findings could suggest the need to extend strict follow-up beyond the 2-year landmark, particularly for those patients with known risk factors [9, 15]. Our results confirm the positive association between CIS, NMIBC, positive ureteral margin and metachronous UTUC, while other clinical determinants emerged, such as younger age at RC and a positive smoking history. Compared to the other well-accepted predictors of UUT recurrence, the association between a positive history of BCG exposure and development of metachronous UTUC is reported in fewer cohorts [7, 16–18]. The higher rates of BCG exposures in the RC + RNU cohort suggests a greater proportion of BCG-unresponsive cases, potentially reflecting more aggressive tumor biology [19, 20]. A pN + status at RC was more frequent in the RC alone group. This pattern is consistent with the poorer prognosis of pN + disease, which may reduce the time window to observe metachronous UUT recurrence. Most UTUCs were found to have adverse pathologic features at the time of RNU, with a predominance of muscle-invasive disease and a substantial proportion of lymph node-positive disease. The high prevalence of adverse pathological characteristics can be partially explained by the fact that only metachronous UTUC managed with RNU were included in the present study. Nonetheless, the predominance of locally advanced disease may reflect inadequate standardization of surveillance protocols, reliance on non-contrast studies in patients with compromised renal function yielding lower diagnostic sensitivity, limited accuracy of cytological examination, and the tendency for UTUC to manifest later in the post-cystectomy period when monitoring intensity has typically been reduced.

Both in the matched and unmatched cohorts, prognosis following RNU after RC was notably poor. These findings underscore the aggressive biological behavior of metachronous UTUC following RC for bladder cancer. To date, only few retrospective studies reported poor survival after RNU in patients previously treated with RC [21, 22]. UTUC in post-cystectomy patients is typically diagnosed following symptomatic presentation–namely hematuria, flank pain, or pyelonephritis–which often reflects advanced overlooked disease [14]. Nonetheless, the detection of asymptomatic UTUC is associated with a 30% reduction of mortality compared to symptomatic presentation [23]. Early detection of an UUT recurrence could not only minimize cancer-related mortality, but also reduce all-cause deaths and improve quality of life, by avoiding radical management in favour of kidney-sparing strategies [24]. Taken together, our results suggest that a one-size-fits-all follow-up protocol may be insufficient after RC. Patients carrying multiple risk factors—namely concomitant CIS, history of recurrent NMIBC, positive ureteral margins at RC, younger age, and BCG-failure—represent a high-risk subgroup warranting intensified and prolonged UUT surveillance, potentially with semi-annual CT urography beyond the standard three-year window. Moreover, our results corroborate the reported worse survival outcomes of variant histology (VH) in UTUC patients at RNU [25]. Nonetheless, whether these prognostic implications should translate into prompt adjuvant therapy or intensified follow-up remains unclear, particularly for the already vulnerable population of RC patients.

Several limitations of our study must be considered. First, the retrospective nature and relatively small sample size of the RC + RNU cohort could have influenced the results or masked significant associations. Second, although PSM allowed us to obtain similar and comparable study groups, other relevant confounders might not have been evaluated. Third, since the timing of RNU was not uniform and follow-up duration significantly differed between the two groups, survival outcomes might have been affected by time-dependent biases such as lead-time bias and immortal time bias. Lastly, relevant information, such as clinical presentation, time from UTUC diagnosis to surgical treatment and adopted surveillance schedules, were not available.

## Conclusions

In the present multicentric, propensity score-matched analysis, approximately 5% of patients undergoing RC with curative intent for BC were subsequently submitted to RNU for a metachronous UTUC. Moreover, most of the UUT recurrences were diagnosed more than two years after RC. Established risk factors, such as concomitant CIS, NMIBC, and positive ureteral margins were confirmed as significant predictors of UUT recurrence, while younger age, BCG-failure and smoking history emerged as possible additional clinical determinants. Even after adequate matching for baseline characteristics, patients who developed a metachronous UTUC treated with RNU after RC exhibited markedly worse survival outcomes compared to the RC-alone cohort. The high prevalence of locally-advanced disease at RNU, together with the impaired survival outcomes, suggest that current surveillance protocol might be insufficient to detect early stage UTUC, particularly for high-risk cases carrying multiple risk factors. Our findings corroborate the need for standardized, risk-stratified, stringent and long-term follow-up of the remnant urinary tract following RC. In the future, large prospective studies are required to further explore the optimal surveillance strategy of BC survivors.

## Supplementary Information

Below is the link to the electronic supplementary material.

Supplementary file1 (DOCX 110 KB)

Supplementary file2 (DOCX 105 KB)

Supplementary file3 (DOCX 20 KB)

Supplementary file4 (DOCX 15 KB)

## Data Availability

Additional data is partially available upon request to the corresponding author.
