# Supplementary material for: Nephroureterectomy for upper tract urothelial carcinoma recurrence in bladder cancer patients treated with radical cystectomy: a multicentric propensity score matched analysis on predictors, practice patterns and survival outcomes
Source: World J Urol. 2026 Jun 10;44(1):419. doi: 10.1007/s00345-026-06520-z (PMC13253654; doi:10.1007/s00345-026-06520-z)
Supplement: Supplementary file 1 — Supplementary file1 (DOCX 110 KB) [file 345_2026_6520_MOESM1_ESM.docx]

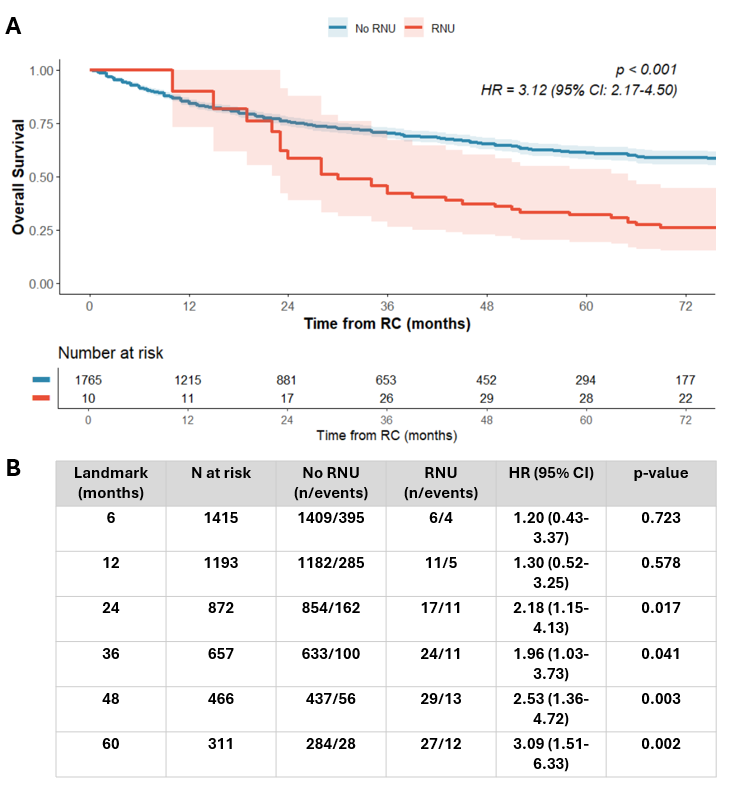


**Supplementary figure 1. A**: Simon-Makuch survival analysis comparing overall survival (OS) distributions between the RC alone group and the RC + RNU group in the unmatched cohort. Restricted mean survival time (RMST) since RC was 101.5 months (SE = 2.88) for the no-RNU group and 50.7 months (SE = 8.25) for the RNU group. Cox proportional hazards regression with RNU as a time-dependent covariate showed a significantly higher risk of all-cause death in patients requiring RNU (HR = 3.12, 95% CI: 2.17-4.50, p < 0.001); **B**: Landmark analysis for OS according to RNU status (unmatched cohort).
