# Supplementary material for: Nephroureterectomy for upper tract urothelial carcinoma recurrence in bladder cancer patients treated with radical cystectomy: a multicentric propensity score matched analysis on predictors, practice patterns and survival outcomes
Source: World J Urol. 2026 Jun 10;44(1):419. doi: 10.1007/s00345-026-06520-z (PMC13253654; doi:10.1007/s00345-026-06520-z)
Supplement: Supplementary file 2 — Supplementary file2 (DOCX 105 KB) [file 345_2026_6520_MOESM2_ESM.docx]

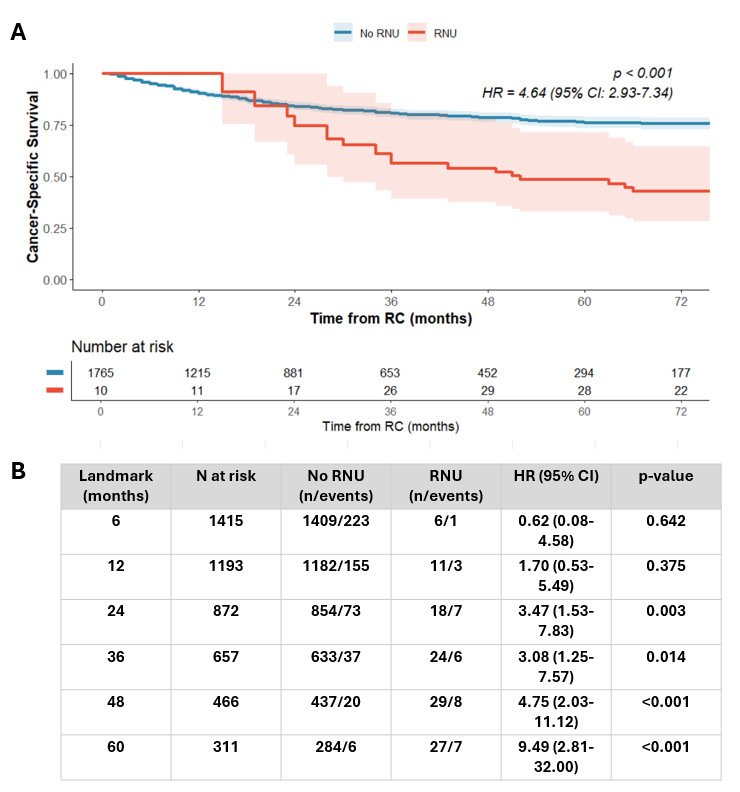


**Supplementary figure 2. A**: Simon-Makuch survival analysis comparing Cancer Specific Survival (CSS) distributions between the RC alone group and the RC + RNU group in the unmatched cohort. RMST was 130.6 months (SE = 2.25) for the no-RNU group and 73.4 months (SE = 10.90) for the RNU group. Cox proportional hazard regression with RNU as a time-dependent covariate showed significantly higher cancer-related risk of death in patients who underwent RNU (HR: 4.64, 95%CI 2.93 - 7.34, p<0.001).; **B**: Landmark analysis for CSS according to RNU status (unmatched cohort).
