# Supplementary material for: Nephroureterectomy for upper tract urothelial carcinoma recurrence in bladder cancer patients treated with radical cystectomy: a multicentric propensity score matched analysis on predictors, practice patterns and survival outcomes
Source: World J Urol. 2026 Jun 10;44(1):419. doi: 10.1007/s00345-026-06520-z (PMC13253654; doi:10.1007/s00345-026-06520-z)
Supplement: Supplementary file 3 — Supplementary file3 (DOCX 20 KB) [file 345_2026_6520_MOESM3_ESM.docx]

**Supplementary Table 1**. General characteristics of the study population prior to Propensity Score Matching. **RC**: Radical Cystectomy; **RNU**: Radical Nephroureterectomy; **IQR**: Interquartile Range; **BMI**: Body Mass Index; **BCG**: Bacillus Calmette-Guérin; **MIBC**: Muscle-Invasive Bladder Cancer; **TURBT**: Transurethral Resection of Bladder Tumor; CIS: Carcinoma In Situ.

| **Variable** | **RC only(n=1,719)** | **RC + RNU (n=85)** | **p-value** |
| --- | --- | --- | --- |
| **Age, median (IQR)** | 69.0 (62.0-75.0) | 68.6 (60.8-72.6) | 0.087 |
| **Gender, n (%)** |  |  | 0.930 |
| Male | 1,358 (79.0%) | 68 (80.0%) |  |
| Female | 361 (21.0%) | 17 (20.0%) |  |
| **BMI, median (IQR)** | 25.7 (23.5-28.0) | 26.4 (24.0-29.7) | 0.135 |
| **Charlson Comorbidity Index, median (IQR)** | 4.0 (2.0-5.0) | 5.0 (4.0-6.0) | <0.001 |
| **Smoking Status, n (%)** |  |  | 0.013 |
| No | 685 (39.8%) | 19 (22.4%) |  |
| Active/Former | 1,034 (60.2%) | 66 (77.6%) |  |
| **BCG Exposure, n (%)** |  |  | <0.001 |
| No | 1,402 (81.6%) | 41 (48.2%) |  |
| Yes | 317 (18.4%) | 44 (51.8%) |  |
| **MIBC at TURBT, n (%)** |  |  | <0.001 |
| No | 541 (31.5%) | 57 (67.1%) |  |
| Yes | 1,178 (68.5%) | 28 (32.9%) |  |
| **Clinical T Stage, n (%)** |  |  | <0.001 |
| cT0 | 9 (0.5%) | 0 (0.0%) |  |
| cT1 | 385 (22.4%) | 34 (40.0%) |  |
| cT2 | 1,140 (66.3%) | 32 (37.6%) |  |
| cT3 | 26 (1.5%) | 2 (2.4%) |  |
| cT4 | 12 (0.7%) | 4 (4.7%) |  |
| cTis | 47 (2.7%) | 8 (9.4%) |  |
| cTa | 100 (5.8%) | 5 (5.9%) |  |
| **Concomitant CIS, n (%)** |  |  | <0.001 |
| No | 1,411 (82.1%) | 48 (56.5%) |  |
| Yes | 308 (17.9%) | 37 (43.5%) |  |
| **Clinical N Stage, n (%)** |  |  | 0.155 |
| cN0 | 1,580 (91.9%) | 84 (98.8%) |  |
| cN+ | 139 (8.1%) | 1 (1.2%) |  |
| **Neoadjuvant Systemic Treatment, n (%)** |  |  | 0.664 |
| No | 1,373 (79.9%) | 70 (82.4%) |  |
| Yes | 346 (20.1%) | 15 (17.6%) |  |
| **RC approach, n (%)** |  |  | 0.821 |
| Open | 1106 (64.4%) | 54 (63.5%) |  |
| Rob/Lap | 613 (35.6%) | 31 (36.5%) |  |
| **Urinary Diversion, n (%)** |  |  | <0.001 |
| Ileal Conduit | 836 (48.6%) | 55 (64.7%) |  |
| Neobladder | 579 (33.7%) | 22 (25.9%) |  |
| Cutaneous Ureterostomy | 304 (17.7%) | 6 (7.1%) |  |
| Ileal Pouch | 0 (0.0) | 2 (2.4) |  |
| **Pathologic T Stage, n (%)** |  |  | <0.001 |
| pT0 | 361 (21.0%) | 5 (5.9%) |  |
| pT1 | 141 (8.2%) | 22 (25.9%) |  |
| pT2 | 289 (16.8%) | 21 (24.7%) |  |
| pT3 | 488 (28.4%) | 8 (9.4%) |  |
| pT4 | 199 (11.6%) | 7 (8.2%) |  |
| pTis | 173 (10.1%) | 18 (21.2%) |  |
| pTa | 68 (4.0%) | 4 (4.7%) |  |
| **MIBC at RC, n (%)** |  |  | 0.012 |
| No | 743 (43.2%) | 49 (57.6%) |  |
| Yes | 976 (56.8%) | 36 (42.4%) |  |
| **Pathologic N Stage, n (%)** |  |  | <0.001 |
| pN0 | 1,383 (80.5%) | 83 (97.6%) |  |
| pN+ | 311 (18.1%) | 2 (2.4%) |  |
| pNx | 25 (1.5%) | 0 (0.0%) |  |
| **CIS at RC, n (%)** |  |  | 0.005 |
| No | 1,224 (71.2%) | 47 (55.3%) |  |
| Yes | 495 (28.8%) | 38 (44.7%) |  |
| **Variant Histology at RC, n (%)** |  |  | 0.171 |
| No | 1,576 (91.7%) | 74 (87.1%) |  |
| Yes | 143 (8.3%) | 11 (12.9%) |  |
