# Supplementary material for: Nephroureterectomy for upper tract urothelial carcinoma recurrence in bladder cancer patients treated with radical cystectomy: a multicentric propensity score matched analysis on predictors, practice patterns and survival outcomes
Source: World J Urol. 2026 Jun 10;44(1):419. doi: 10.1007/s00345-026-06520-z (PMC13253654; doi:10.1007/s00345-026-06520-z)
Supplement: Supplementary file 4 — Supplementary file4 (DOCX 15 KB) [file 345_2026_6520_MOESM4_ESM.docx]

**Table 4**. Multivariable Cox Regression Analysis exploring independent UTUC-related predictors of Overall Survival (OS) and Cancer Specific Survival (CSS) in the RC + RNU Subgroup. **OS**: Overall Survival; **CSS**: Cancer-Specific Survival; **HR**: Hazard Ratio; **CI**: Confidence Interval; **RNU**: Radical Nephroureterectomy; **CHT**: Chemotherapy; **VH**: Variant Histology; **CIS**: Carcinoma In Situ.

|  | **OS** | | | **CSS** | | |
| --- | --- | --- | --- | --- | --- | --- |
| **Variable** | **HR** | **95% CI** | **p-value** | **HR** | **95% CI** | **p-value** |
| **Time to RNU** | 1.00 | 0.99-1.02 | 0.460 | 1.00 | 0.99-1.02 | 0.662 |
| **Neoadjuvant CHT before RNU** | 0.37 | 0.09-1.65 | 0.195 | 0.40 | 0.08-1.95 | 0.254 |
| **Tumor location** | 0.91 | 0.56-1.48 | 0.709 | 0.83 | 0.43-1.59 | 0.572 |
| **pT stage>pT1** | 1.54 | 0.68-3.49 | 0.299 | 4.20 | 0.91-19.33 | 0.066 |
| **pN+** | 2.19 | 1.00-6.72 | 0.049 | 3.96 | 1.54-10.14 | 0.004 |
| **VH RNU** | 4.12 | 1.56-10.89 | 0.004 | 4.30 | 1.29-14.33 | 0.017 |
| **CIS RNU** | 0.65 | 0.31-1.36 | 0.257 | 0.78 | 0.31-1.98 | 0.605 |
